# Expert Witnesses

**Published:** 1898-03-19

**Authors:** 


### The Hospital

A Journal of

The Medical Sciences and Hospital Administration.

Vol. XXIII.—No. 59?.] Edited by Sir Henry Burdett, K.C.B., and Solomon C. Smith, M.D., M.R.C.P. [March 19, 1898.

### Expert Witnesses.

A Bill has been introduced into the Legislature of the State of New York for the purpose of regulating the employment of "expert witnesses" in trials, and the amounts which should be paid to them. Many Bills are introduced at Westminster with no expectation that they will ever reach the stage of enactment, but merely in order to propitiate some section of the supporters of the introducer; and it is possible that the same artifice may not be unknown on the other side of the Atlantic. Be this as it may, and although the American Bill is described as springing from so questionable a source as the "Homœopathic State Medical Society," there can be no doubt that it points to the existence of a real evil, and one which, not many years ago, prevailed somewhat extensively among ourselves. A late witty judge is well known to have classified false witnesses under the three heads of "liars, d——d liars, and experts"; and it is quite certain that an element of partisanship, of desire to do their best for the litigant who had employed them, used seldom to be absent from the testimony of the latter. In many instances, there would be a certain amount of probability in favour of each of two different or even opposite views, and these views would each be presented by men whose opinions were of recognised value, but between whose conflicting statements it would be impossible for a jury to decide by any other process than that of tossing up. A lady, for example, received an accidental blow on her right eye from the whip of a passing coachman, and sued the coachman's master for damages. Experts examined her, and it was found that she had an early stage of cataract in both eyes, but more advanced in the right than in the left. On this both sides were agreed. The plaintiff's expert, however, maintained that the blow had either been the exciting cause of the cataract in the right eye, or, at all events, the cause why it had made more progress in this eye than in its fellow; while the defendant's expert maintained, with equal force and with equal show of reason, that both cataracts were entirely independent of the trivial injury, and that a more advanced condition in one eye than in the other was an ordinary incident of the affection. Both experts, if they had been in the hands of counsel who knew how to cross-examine them, would have been made to confess that their respective views were hypothetical, and that there was about as much to be said in favour of one as of the other. Manner, dogmatism, reputation, would have much influence upon the decision; and, in these circumstances, not so many years ago, there arose in London a class of men who systematically laid themselves out for being medical witnesses, and who were to be found, on one side or the other, in almost every trial between a railway company and an injured passenger. The conflicts of testimony thus produced were often scandalous enough; but they were seldom reported with any length or fulness, and therefore did less than might have been expected to lower the dignity and diminish the credit of the profession.

The proposal of the American Bill is that, in any criminal trial the decision of which is likely to turn upon medical evidence, the Court, on the application either of the prosecution or of the defence, may appoint a sufficient number of experts, not more than five nor less than three, to give skilled testimony, each of whom will be paid from 10 dols. to 100 dols. a-day for his services. It has often been proposed in this country, although we are not aware that any attempt at legislation upon the subject has been made, that the presiding judge should have power to call in an expert, whose function should be rather that of a medical assessor, and who should advise the jury as to the value and bearing of evidence given by the retained witnesses, whom it would obviously be impossible to prevent either side from calling whenever they might think their respective cases would be strengthened by doing so. There would be to some extent a precedent for such a course in the arrangement by which nautical assessors now sit with the presiding judge of the Admiralty Court in cases which involve questions of seamanship; and it is probable that the presence of such assessors would tend to restrain the imaginations of witnesses who might other wise yield to the temptation of eking out their facts by embroidery. Lawyers, we believe, are accustomed to maintain that unskilled persons are competent judges of the value of skilled testimony; but everyone who has given such testimony is well aware that this view is wholly erroneous. Even counsel themselves, even the ornaments of the judicial bench, are sometimes manifestly unable to grasp the meaning or estimate the bearing and weight of expert evidence; and, if not they, why or how the jurors? We were once in Court when three or four ophthalmic surgeons had been giving evidence, and when paintings of the fundus of an eye, taken at different times, had been produced and appealed to. The Judge recalled one of the witnesses. "I want to know," he said, "why these pictures, which are said to be representations of the interior of the eye, should be painted of a red colour?" "Because they represent red structures," said the witness. "Oh," replied the Judge, "you may stand down, sir." His Lordship thought the interior of the eye was black, and that the "expert" himself was of the character described in our quotation.

